# From Spark to Strain? Changes in Relationship Satisfaction as a Function of Narcissistic Admiration and Rivalry

**DOI:** 10.1111/jopy.70065

**Published:** 2026-03-26

**Authors:** Gwendolyn Seidman, William J. Chopik

**Affiliations:** ^1^ Michigan State University East Lansing Michigan USA

**Keywords:** growth curve modeling, narcissistic admiration, narcissistic rivalry, PAIRFAM, relationship satisfaction

## Abstract

**Objective:**

Narcissism is often proposed to drive attraction early in relationships but ultimately leads to poor long‐term outcomes that are distressing to narcissistic individuals' partners. We examined changes in relationship satisfaction over a six‐year period as a function of narcissistic admiration and narcissistic rivalry.

**Method:**

Data came from 5869 romantic dyads and a subsample of 533 early relationships within their first year. Participants were part of the German Family Panel (pairfam). Respondents completed the short form of the Narcissistic Admiration and Rivalry Questionnaire. For 6 years, both respondents and their partners completed a single‐item relationship satisfaction measure.

**Results:**

Both one's own and one's partner's narcissistic rivalry were associated with lower satisfaction in the full sample, but not the new relationships sample. Narcissistic admiration was not significantly associated with satisfaction. Contrary to predictions, neither partner's narcissism moderated relationship satisfaction changes in either sample.

**Conclusions:**

Despite its association with lower baseline satisfaction, narcissistic rivalry failed to moderate longitudinal changes in relationship satisfaction. These findings challenge the assumption of a linear cost to narcissism over time and highlight the need to investigate alternative time scales or heterogeneous relationship outcomes.

Grandiose narcissism involves inflated self‐perceptions, self‐centeredness, and entitlement (e.g., John and Robins 1994; Morf and Rhodewalt 2001). As a result, narcissism is associated with negative relationship outcomes in long‐term, established romantic relationships. However, although ostensibly these characteristics might spell doom for relationships, narcissism is often associated with *positive* impressions at first acquaintance and success in the early stages of romantic relationships (e.g., Dufner et al. 2013; Friedman et al. 2006).

Existing frameworks characterizing narcissism have been helpful in explaining these differential outcomes for narcissistic individuals. The Narcissistic Admiration and Rivalry Concept (NARC; Back et al. 2013), which describes two dimensions of narcissism, one admiration‐seeking and the other antagonistic and rivalrous, has been useful in explaining differential short and long‐term relationship outcomes (Wurst et al. 2017). Other, related theories describe how the behaviors displayed by narcissistic individuals may change over time and affect the impressions formed by their romantic interests or partners (Brunell and Campbell 2011; Campbell et al. 2006). Collectively, these theories suggest that partners of narcissistic individuals may experience particularly satisfying relationships initially (partially because of narcissistic individuals' charming tendencies) but then experience especially steep declines in satisfaction over time (partially because of narcissistic individuals' antagonistic tendencies).

Despite this suggestion and theorizing, research findings linking narcissism and relationship satisfaction have been mixed (Casale et al. 2020; Lamkin et al. 2015; Ye et al. 2016), and longitudinal data on this association are limited (Kislev 2023; Lavner et al. 2016). The aforementioned frameworks may be especially useful for understanding long‐term changes in the relationship satisfaction of people with highly narcissistic partners. The present study examined changes in relationship satisfaction in 5869 romantic dyads over a period of six years as a function of one partner's levels of narcissistic admiration and narcissistic rivalry. We also examined whether these processes varied depending on how long the couples have been together.

## 1 Narcissism and Short vs. Long‐Term Relationship Outcomes

Narcissism involves grandiose self‐views, vanity, self‐centeredness, and a sense of entitlement (Morf and Rhodewalt 2001). To maintain their grandiose self‐perceptions, narcissistic individuals use both intrapersonal strategies (e.g., taking credit for positive outcomes, viewing oneself as superior to other people) and interpersonal strategies (e.g., manipulating others, seeking admiration over liking, striving for power; see Morf and Rhodewalt 2001 for review). Thus, narcissistic individuals strive to be admired but also behave in ways that are destructive for their interpersonal relationships.

One conceptualization of narcissism that helps to explain its dual nature is the Narcissistic Admiration and Rivalry Concept (NARC; Back et al. 2013; Back 2018). According to the NARC model, narcissistic individuals have two strategies for maintaining their grandiose self‐perceptions. The first strategy, narcissistic admiration, involves seeking admiration from others by being charming and asserting one's unique and special status. The second strategy, narcissistic rivalry, is more antagonistic and interpersonally destructive. It involves devaluing others and demonstrating superiority over them. Narcissistic admiration is associated with positive characteristics, such as higher self‐esteem, agentic interpersonal behavior, and the use of more constructive conflict strategies. Narcissistic rivalry is associated with negative characteristics, such as lower self‐esteem, greater hostility, less forgiveness, and less communal interpersonal behavior.

Narcissism is an interpersonally destructive strategy in the long‐term but may have short‐term interpersonal advantages. According to Campbell and Campbell's (2009) contextual reinforcement model, narcissism provides an *advantage* during early, emerging relationships when narcissistic individuals' charm and agentic qualities are attractive to others, but a *disadvantage* in enduring relationships. Brunell and Campbell (2011) explain that this may aid in understanding the appeal of narcissistic individuals from their romantic partners' perspective. According to the “Chocolate Cake Model” of narcissism, romantic relationships with narcissistic individuals are like chocolate cake: pleasurable in the short‐term but unhealthy in the long‐term (Brunell and Campbell 2011). This is even true in friendships: within friendship groups, narcissism may have early advantages that later disappear or reverse. When groups of strangers engaged in a getting‐acquainted task, narcissism was associated with greater likability. But as these groups continued to meet, the direction of the association reversed, and narcissism was associated with *less* liking (Paulhus 1998). In a study of friendship networks, narcissism was associated with greater popularity, and, although people typically like friends more as they interact more, this effect is blunted among narcissistic individuals (Czarna et al. 2016).

What are narcissistic individuals doing at various points of the relationship cycle that explain why their initially positive impressions sour over time? The NARC framework is especially useful for understanding the short‐term advantages and long‐term disadvantages of narcissism in romantic relationships. In zero‐acquaintance settings, narcissistic individuals are well‐liked and successful in initiating relationships. Observers have more positive impressions of narcissistic individuals when exposed to thin slices of their behavior (Friedman et al. 2006; Oltmanns et al. 2004). Particularly relevant to the current study, narcissism is associated with greater attractiveness in short‐term mating and romantic relationship initiation contexts (Dufner et al. 2013; Jauk et al. 2016). Further, Wurst et al. (2017) demonstrated that narcissistic admiration was associated with greater likability and attractiveness during zero‐acquaintance interactions and with greater short‐term mate appeal as rated by peers.

As relationships develop and enter the longer‐term, enduring zone, the more negative characteristics of narcissistic individuals create difficulties. They tend to be manipulative and lack commitment to their partners (Campbell and Foster 2002; Foster 2008). They respond with greater aggression during relationship conflicts (Keller et al. 2014) and devalue partners both during and after conflict (Peterson and DeHart 2014). In studies examining long‐term relationships, narcissistic rivalry is associated with numerous relationship difficulties: less commitment, more conflict, less conciliatory and more dysfunctional reactions to transgressions, and more negative perceptions of romantic partners (Wurst et al. 2017). Additional studies have demonstrated that narcissistic rivalry is associated with a variety of negative outcomes and behaviors in long‐term relationships, including less commitment, less respect toward one's partner, less positive bias in perceptions of partner attractiveness, and the use of negative influence tactics (Sauls et al. 2019; Seidman et al. 2023; Vrabel et al. 2021; Zeigler‐Hill et al. 2020). In a longitudinal study of new friendships among unacquainted undergraduates, Leckelt et al. (2020) found that agentic behaviors increased popularity over time, even when trait‐like narcissistic admiration and rivalry were not directly related to being liked. Further, popularity increased among those high in narcissistic admiration but decreased among those high in narcissistic rivalry (who often engaged in antagonistic behaviors). Collectively, these studies provide partial support for the notion that narcissistic admiration would help, and narcissistic rivalry would hinder, likability over time.

## 2 Narcissism and Trajectories of Relationship Satisfaction

Several studies have examined associations between individual (i.e., actor effects) narcissism or being partnered with a narcissist (i.e., partner effects) and relationship satisfaction. Generally, both actor and partner narcissism are associated with lower satisfaction, but these effects sometimes vary by gender. In terms of actor effects, Casale et al. (2020) and Ye et al. (2016) found that both men's and women's grandiose narcissism was related to lower relationship satisfaction. However, Gewirtz‐Meydan and Finzi‐Dottan (2018) found that only men's, and not women's, narcissism predicted one's own relationship satisfaction. In terms of partner effects, Ye et al. (2016) found that both men's and women's narcissism were related to lower satisfaction for their partners. However, Casale et al. (2020) and Gewirtz‐Meydan and Finzi‐Dottan (2018) only observed this association for the effect of female narcissism on male partners' satisfaction. Finally, Lamkin et al. (2015) did not find any overall actor or partner effects of narcissism on relationship adjustment (a combination of satisfaction, affectionate expression, and cohesion) among undergraduate dating couples. In sum, higher levels of both actor and partner narcissism are associated with lower relationship satisfaction cross‐sectionally, although there is some ambiguity over whether the results are more pronounced among men or women.

Having said that, longitudinal work on the association between narcissism and relationship satisfaction is limited. However, in making inferences about how narcissism might affect relationships over time, it can be useful to examine how these effects are different among those in shorter‐ and longer‐term romantic relationships and among newlyweds followed over the early years of marriage. In the aforementioned cross‐sectional study of dating couples, Lamkin et al. (2015) found that relationship adjustment was lower among long‐term couples if both partners were high in narcissism, particularly elements related to entitlement/exploitativeness. In a longitudinal study of newlyweds during the first 4 years of marriage, Lavner et al. (2016) also found that entitlement/exploitativeness predicted declines in wives' own marital satisfaction over time, but husbands' narcissism had few effects on either their own or their wives' marital satisfaction. Given the disparate findings of narcissism's effects on short‐ and long‐term relationships, do these longitudinal patterns depend on if relationships were in their initial or established stages? Typically, such rare moderation analyses have yielded mixed results, but many studies are based on relatively young (e.g., undergraduate, newlywed) samples who often can *only* be in emerging relationships.

Previous studies examining how narcissism relates to satisfaction over time have also rarely distinguished between narcissistic rivalry and admiration in the NARC framework, even though doing so has been invaluable at characterizing outcomes across the relationship cycle. Cross‐sectionally, narcissistic admiration is associated with higher, and narcissistic rivalry with lower, relationship satisfaction (e.g., Wurst et al. 2017; Zeigler‐Hill et al. 2024). The only study to examine *longitudinal* changes in relationship satisfaction as a function of changes in the NARC dimensions was Kislev's (2023) analysis of an earlier version of the Pairfam data (which we use in this study). Kislev (2023) found that increases in one's own narcissistic rivalry over a two‐year period were associated with declines in relationship satisfaction for both men and women. Increases in narcissistic admiration were associated with increasing satisfaction for women, but not men (Kislev 2023). Although valuable in integrating the NARC model into understanding how narcissism affects relationship satisfaction over time, that paper was limited in a few ways. For example, adopting an individual approach neglects how the partners of narcissists feel about their relationship (a crucial theoretical consideration in the work we described above). Further, much of the existing theory makes predictions about how baseline narcissism affects changes in relationship satisfaction over time (such that it might be particularly harmful as the relationship progresses). In the current study, we more deliberately adopted a dyadic approach to examine how being partnered with someone high in narcissistic admiration or rivalry was associated with changes in relationship satisfaction over time. We also examined whether these effects differed between relationships that are in the emerging versus more established stage.

## 3 The Present Study

Generally, romantic relationship satisfaction tends to decline over time, but these effects are heterogenous across couples (e.g., Bühler et al. 2021). Prior theories and research on narcissism discussed previously suggest that narcissism is likely to moderate changes in satisfaction. Specifically, these theories suggest that partners of narcissistic individuals should experience steeper declines in relationship satisfaction than those whose partners are less narcissistic. This should be especially true when examining relationships that are new. Specifically, romantic partners may be initially captivated by the charisma a narcissistic partner displays. But this initial infatuation likely fades as some of the more problematic aspects of narcissism rear their head in people's relationships. We operationalized this idea by hypothesizing that people partnered with narcissistic individuals may show elevated initial satisfaction with steeper declines over time, compared to people partnered with those lower in narcissism.

We analyzed longitudinal data on romantic couples' relationship satisfaction assessed in both partners annually over a period of up to six years. We used dyadic growth curve analyses based on the actor‐partner interdependence model (APIM; Kenny et al. 2006). This allowed us to test hypotheses regarding changes in one's own (actor's) and one's partner's satisfaction as a function of one's own (actor's) levels of narcissistic admiration and narcissistic rivalry.^1^

We tested the following hypotheses:

*Relationship satisfaction will decline over time. Specifically, the linear effect of time will be negative and significant*.

*Actor narcissistic admiration will be positively associated with partner satisfaction in new relationships (in relationships of* 1 *year or less). Specifically, we expected narcissistic admiration to be associated with greater partner satisfaction; however, the magnitude of this association will be higher in newer relative to more established relationships*.

*Higher levels of actor narcissistic rivalry will be associated with lower partner satisfaction and steeper declines in partner satisfaction over time. We expected this decline to be especially strong in new relationships. Specifically, the linear effect of time will be particularly negative among those with partners higher in narcissistic rivalry. We further expected this rivalry × time interaction to be stronger in newer relative to more established relationships*.^2^

We tested the following research question for which we did not have any explicit hypotheses:

RQ1: How are actor narcissistic admiration and narcissistic rivalry associated with actor initial satisfaction in new relationships and with changes in satisfaction over time? This research question primarily focuses on “actor” effects (similar to those seen in Kislev (2023), but conducted in the context of a dyadic growth curve analysis).

## 1 Method

### 1.1 Participants and Procedure

Participants were from the Panel Analysis of Intimate Relationships and Family Dynamics, release 14.2 (PAIRFAM; Brüderl et al. 2024).^3^ See Huinink et al. (2011) for a detailed description of study procedures. Pairfam surveyed a nationally representative sample of over 12,000 German adults born between 1971–1973, 1981–1983, and 1991–1993, with data collected annually beginning in 2008 for 14 consecutive years. A cohort born in 2001–2003 was added in wave 11. Data were also collected from respondents' romantic partners if applicable, although the questions asked of respondents and their partners varied across waves. We used data from waves 9 through 14 because Wave 9 was the first time narcissism measures were included in the study. The primary respondent is referred to as the anchor and their partner is referred to as the partner.

We selected all anchors who were involved in a romantic relationship with the same partner for at least two timepoints during Waves 9 through 13. If a participant had more than one partner during that period, we selected data from the partner with whom the participant had the longest relationship. We defined Time 1 as the first year in which the participant was in a relationship with that particular partner and provided data on the primary variables of interest here. Thus, for most participants this was Wave 9, and for most participants in the newer cohort, this was Wave 11.

Our sample contained 5869 dyads (anchors were 57.7% female, partners were 57.4% male). The average age of anchors was 32.78 (SD = 8.83) and the average age of partners was 33.80 (SD = 9.96). The average relationship length was 100.63 months (SD = 94.51).

To analyze the trajectory of satisfaction for a subset of individuals who were in new relationships, we selected the subsample of the broader 5869 dyad sample whose relationships were less than 13 months long at the time of the survey. This yielded 533 dyads (anchors were 52.5% female, partners were 50.7% male). The average age of anchors was 25.99 (SD = 8.17), the average age of partners was 26.32 (SD = 9.12), and the average relationship length was 6.52 months (SD = 3.44).

We used Ackerman and Kenny's (2016) APIMPowerR shinyapp to evaluate the smallest possible effects we could detect with our samples. Among the newer relationships sample (the smaller of the two analytic samples; *N* = 533 couples), we were able to detect associations as small as *r* = 0.162 with 80% power at alpha = 0.05 (assuming correlations of *r* = 0.30 among errors between partners). The main sample (*N* = 5869) afforded us more statistical power, enabling us to detect associations as small as *r* = 0.04.

### 1.2 Measures

#### 1.2.1 Narcissism

Anchors completed narcissism measures beginning in Wave 9, and then again every 2 years. Anchors completed the short form of the Narcissistic Admiration and Rivalry Questionnaire (NARQ‐S; Back et al. 2013). The NARQ‐S contains three items assessing narcissistic admiration and three items assessing narcissistic rivalry. Participants rated each item from 1 (*not at all*) to 5 (*absolutely*). For our analyses, we used the anchors' first NARQ‐S measurement (either Wave 9 or 11, depending upon when they joined the panel) and modeled narcissistic admiration (All couples: *M* = 1.87, SD = 0.86, *α* = 0.778, New relationships: *M* = 2.12, SD = 0.90, *α* = 0.756) and narcissistic rivalry (All couples: *M* = 1.53, SD = 0.65, *α* = 0.641, New relationships: *M* = 1.66, SD = 0.70, *α* = 0.643) as time‐invariant predictors. Narcissism data were collected only among anchors, although relationship satisfaction (see next) was collected from both anchors and their partners.

#### 1.2.2 Relationship Satisfaction

At all timepoints (waves 9, 10, 11, 12, 13, and 14), both anchors and partners reported their relationship satisfaction. Participants responded to the item “Overall, how satisfied are you with your relationship?” using a scale from 0 (*very dissatisfied*) to 10 (*very satisfied*).

## 2 Results

### 2.1 Preliminary Analyses

Table 1 displays bivariate intercorrelations between predictor variables. There was a moderate positive correlation between relationship length and age in the full sample and a small correlation in the new relationships sample (i.e., relationships younger than 13 months). The two narcissism dimensions were correlated about 0.47–0.51 in both samples. In the full sample, both narcissism dimensions were lower among younger adults and those in younger relationships. In the new relationships sample, both narcissism dimensions were lower among younger adults, and narcissistic rivalry was associated with longer relationships (potentially a selection effect in that relationships with highly rivalrous people do not last as long).

**TABLE 1:** Intercorrelations between predictor variables for full sample and new relationships sample.

	1.	2.	3.	4.
1. Relationship length (months)		**0.655** ***	**−0.113** ***	**−0.142** ***
2. Age	0.158***		**−0.091** ***	**−0.123** ***
3. Narcissistic Rivalry	0.062***	−0.094***		**0.508** ***
4. Narcissistic Admiration	0.034	−0.079***	0.474***	

*Note:* Correlations on the upper diagonal in bold text are for the full sample; correlations in regular text on the lower diagonal are for the new relationships sample (relationship length < 13 months).

*** *p* < 0.001.

### 2.2 Analytic Plan

We used dyadic growth curve models for distinguishable dyads (Kenny et al. 2006; Nestler et al. 2015) to test associations between narcissism dimensions and relationship satisfaction changes over time. Because narcissism data was only collected for anchors, we used anchor status (i.e., whether the respondent is the anchor or the partner) as the distinguishing variable. This approach allowed us to estimate both actor effects (associations between one partner's narcissism and their own satisfaction) and partner effects (associations between one partner's narcissism and their partner's satisfaction). Predictors included anchor status (anchor = −1, partner = 1), time (i.e., wave), anchor's narcissistic admiration, and anchor's narcissistic rivalry. Control variables were anchor sex (0 = male, 1 = female), anchor age, and relationship length (in months). All quantitative predictor variables were mean‐centered.

To test if the NARQ dimensions moderated the changes in satisfaction over time, we included multiplicative interactions between time and each actor/partner NARQ dimension. To test if any effects differed for anchors and partners, we also interacted anchor status with narcissism, time, and narcissism × time. A significant three‐way anchor status × time × narcissism interaction would indicate that the actor and partner effects of narcissism on relationship satisfaction changes differ from each other. To test potential suppression effects, we first ran two separate models (each one with only one of the two NARQ subscales) and then ran the model that included both NARQ subscales simultaneously. Results were similar in both analyses, so we only present results from this latter full model. These procedures are consistent with our pre‐registered data analysis plan which can be found at https://doi.org/10.17605/OSF.IO/KDW5Q. Syntax and supplemental analyses are available at https://osf.io/xmyfu/.

### 2.3 Tests of Primary Hypotheses

Table 2 displays the estimates, standard errors, confidence intervals and significance testing results for the full model for both the entire sample (left panel) and the subset of new relationships (right panel). Consistent with H1, relationship satisfaction decreased over time in both samples. Narcissistic admiration was not associated with satisfaction for either sample, contrary to H2, which predicted that narcissistic admiration would be associated with higher satisfaction among those in new relationships. Narcissistic rivalry (for both partners) was associated with less satisfaction in the full sample, but not the sample of new relationships, providing partial support to H3 (that narcissistic rivalry would be associated with worse relationship satisfaction, albeit it was not stronger in newer relationships relative to more established relationships; also see below for the hypothesized interaction with time). A significant anchor status × narcissistic rivalry interaction for both samples indicated that actor narcissistic (i.e., one's own) rivalry had a larger association with satisfaction than partner narcissistic rivalry (we did not explicitly hypothesize differences between actor and partner effects for the NARC, but this would be predicted by Orth's 2013 observation that shared method/sources will often yield larger associations). A dual intercept model (in which effects are estimated separately for anchors and partners) revealed that, indeed, although both anchor and partner narcissistic rivalry were associated with lower satisfaction, anchor effects (*b* = −0.347, *S*E = 0.037, *p* < 0.001, CI: −0.419, −0.274) were larger than partner effects (*b* = −0.191, SE = 0.044, *p* < 0.001, CI: −0.278, −0.104).

**TABLE 2:** Growth curve models predicting relationship satisfaction in the full sample and new relationships.

Fixed effects	Full sample					New relationships				
	*b*	SE	*t*	*p*	CI	*b*	SE	*t*	*p*	CI
Intercept	8.034	0.028	286.047	< 0.001	7.98, 8.09	8.367	0.074	113.582	< 0.001	8.222, 8.512
Age	−0.041	0.003	−14.706	< 0.001	−0.046, −0.035	−0.026	0.006	−4.317	< 0.001	−0.038, −0.014
Sex	−0.083	0.037	−2.224	0.026	−0.155, −0.010	0.051	0.101	0.501	0.616	−0.148, 0.250
Relationship length	0.001	0.0003	1.979	0.048	0.000005, 0.001	0.0003	0.014	0.021	0.983	−0.028, 0.029
Time	−0.053	0.006	−8.402	< 0.001	−0.065, −0.041	−0.189	0.027	−6.905	< 0.001	−0.243, −0.135
Narcissistic admiration	0.038	0.026	1.498	0.134	−0.012, 0.089	0.031	0.065	0.473	0.637	−0.096, 0.158
Narcissistic rivalry	−0.273	0.034	−7.993	< 0.001	−0.340, 0.206	−0.120	0.083	−1.445	0.149	−0.283, 0.043
Anchor status	0.125	0.012	10.314	< 0.001	0.101, 0.149	0.158	0.031	5.130	< 0.001	0.097, 0.218
Narcissistic admiration × anchor status	0.004	0.017	0.247	0.805	−0.029, 0.037	0.013	0.039	0.326	0.744	−0.065, 0.090
Narcissistic rivalry × anchor status	0.066	0.022	2.960	0.003	0.022, 0.109	0.149	0.050	3.018	0.003	0.052, 0.247
Narcissistic admiration × time	−0.013	0.009	−1.485	0.138	−0.030, 0.004	0.031	0.036	0.850	0.396	−0.040, 0.102
Narcissistic rivalry × time	0.001	0.012	0.117	0.907	−0.022, 0.025	−0.049	0.044	−1.123	0.263	−0.136, 0.037
Anchor status × time	0.005	0.005	1.047	0.295	−0.004, 0.015	0.006	0.020	0.289	0.773	−0.034, 0.046
Anchor status × time × narcissistic admiration	−0.002	0.007	−0.286	0.775	−0.016, 0.011	0.007	0.027	0.263	0.793	−0.047, 0.061
Anchor status × time × narcissistic rivalry	−0.017	0.009	−1.838	0.066	−0.035, −0.001	0.029	0.033	0.875	0.382	−0.036, 0.095
*Random effects*										
Var. anchor intercept	1.740	0.044		< 0.001	1.656, 1.828	1.294	0.122		< 0.001	1.075, 1.557
Var. partner intercept	1.692	0.053		< 0.001	1.591, 1.800	0.983	0.121		< 0.001	0.772, 1.252
Var. anchor slope	0.053	0.005		< 0.001	0.043, 0.064	0.067	0.028		0.018	0.029, 0.153
Var. partner slope	0.049	0.006		< 0.001	0.038, 0.062	0.071	0.034		0.035	0.028, 0.180
Cov. anchor intercept, partner intercept	0.997	0.040		< 0.001	0.918, 1.076	0.700	0.092		< 0.001	0.521, 0.880
Cov. anchor intercept, anchor slope	0.019	0.011		0.083	−0.002, 0.040	0.060	0.044		0.172	−0.026, 0.146
Cov. anchor intercept, partner slope	0.040	0.013		0.003	0.013, 0.066	0.002	0.049		0.966	−0.094, 0.099
Cov. partner intercept, anchor slope	0.092	0.013		< 0.001	0.067, 0.117	0.118	0.042		0.005	0.035, 0.201
Cov. partner intercept, partner slope	0.059	0.013		< 0.001	0.034, 0.084	0.080	0.049		0.099	−0.015, 0.176
Cov. anchor slope, partner slope	0.034	0.005		< 0.001	0.025, 0.043	0.048	0.023		0.041	0.002, 0.093
*Goodness‐of‐fit indices*										
AIC	134,369					10,549				
−2LL	134,343					10,523				

*Note:* New relationships are less than 13 months. Anchor status is coded as −1 for the anchor and 1 for the partner.

Abbreviations: Cov, covariance; Var, variance.

Several two‐way interactions tested whether narcissism dimensions were associated with changes in relationship satisfaction, regardless of anchor status. However, there were very few significant interactions. Specifically, neither NARQ dimension moderated declines in relationship satisfaction, on average. In addition, anchor status largely did not moderate associations between NARQ dimensions and relationship satisfaction (or how it changed over time).

Contrary to H3, which predicted that declines in satisfaction would be steeper for those whose partners scored high in narcissistic rivalry, there was no significant interaction between narcissistic rivalry, time, and anchor status in the new relationship sample. In partial support of H3, this interaction was marginally significant in the full sample. However, when running a dual intercept model, the narcissistic rivalry × time interaction was not significant for either anchors or partners (*p*s > 0.180). This model showed that the marginally significant interaction in the single‐intercept model occurred because anchor narcissistic rivalry was associated (not significantly) with *increasing* satisfaction over time (*b* = 0.018, SE = 0.014, *p* = 0.180, CI: −0.008, 0.045), while partner narcissistic rivalry was associated (not significantly) with *decreasing* satisfaction over time (*b* = −0.016, SE = 0.016, *p* = 0.336, CI: −0.047, 0.016); thus, this interaction found that these two estimates were significantly different from each other despite them not differing from zero.

Finally, age was associated with lower satisfaction. Relationship length (i.e., shorter relationships) and female sex were also associated with lower satisfaction (in the full sample only). Surprisingly, anchors were significantly less satisfied than partners in both samples.^4^

### 2.4 Exploratory Analyses

There are a few different ways to examine whether the effects of anchor/partner narcissism on relationship satisfaction over time vary between short‐ and long‐term relationships.^5^ One alternative way—instead of segmenting a sample of newer relationships—is to formally model relationship length as a continuous moderator in the analyses reported in Table 2. We initially ran the analyses in the full sample and among short‐term relationships as a way of privileging model parsimony (because adding relationship length in this alternate analysis requires estimating four‐way interactions). Nevertheless, we ran an expanded model of what is featured in Table 2 adding multiplicative interactions with relationship length among the full sample.

The results of this model can be found in Table S1. Relationship length rarely moderated the effects reported in Table 2 (nor was the four‐way interaction of narcissistic rivalry × time × anchor status × relationship length significant; *p* = 0.489). However, there were three exceptions: the main effects of (1) time and (2) narcissistic rivalry, and the (3) narcissistic rivalry × time interaction were each moderated by relationship length. Decomposing these effects (at ± one standard deviation for relationship length; ~96 months) revealed that relationship satisfaction declines were more dramatic among newer relationships (*b* = −0.104, SE = 0.010, *p* < 0.001, CI: −0.123, −0.085) compared to more established relationships, for which relationship satisfaction did not significantly decline (*b* = −0.013, SE = 0.008, *p* = 0.120, CI: −0.029, 0.003). The main effect of narcissistic rivalry on relationship satisfaction (on average) was more damaging among more established relationships (*b* = −0.381, SE = 0.055, *p* < 0.001, CI: −0.490, −0.273) compared to newer relationships (*b* = −0.214, SE = 0.045, *p* < 0.001, CI: −0.303, −0.126).

Finally, the two‐way narcissistic rivalry × time interaction was stronger among more established relationships (*b* = 0.041, SE = 0.017, *p* = 0.015, CI: 0.008, 0.074) compared to newer relationships (*b* = −0.030, SE = 0.017, *p* = 0.083, CI: −0.064, 0.004). Among those in established relationships, relationship satisfaction declines were steeper among established couples lower in narcissistic rivalry (*b* = −0.038, SE = 0.013, *p* = 0.003, CI: −0.064, −0.013) compared to established couples higher in narcissistic rivalry (*b* = 0.013, SE = 0.014, *p* = 0.353, CI: −0.014, 0.040). This may seem surprising to some readers at first—that those lower in rivalry would experience *steeper* declines in relationship satisfaction over time among established couples. However, given that narcissistic rivalry has such a strong (negative) main effect on relationship satisfaction, this pattern mostly reflects the fact that couples low in narcissistic rivalry have “further to fall” compared to high narcissistic rivalry couples who had considerably lower relationship satisfaction to begin with. Nevertheless, couples with lower levels of narcissistic rivalry were still more satisfied at the end of the study (see Figure 1). Importantly, this effect was *not* moderated by anchor status, suggesting that these counterintuitive declines are consistent for both anchors and partners.^6^

**FIGURE 1:**
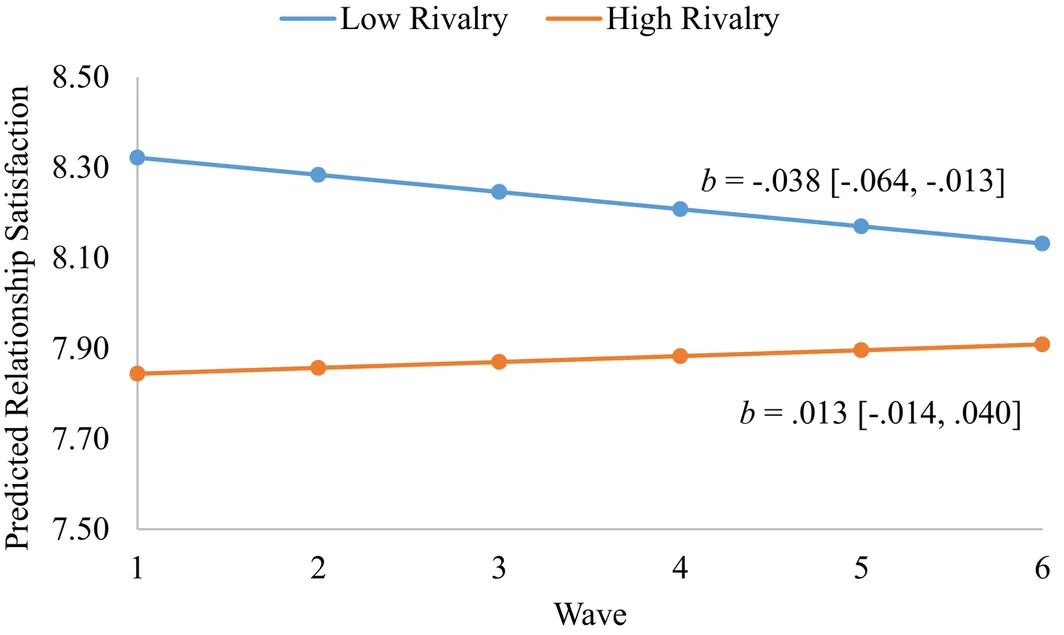
Narcissistic rivalry and changes in relationship satisfaction among established couples.

## 3 Discussion

In this study, we examined whether narcissistic admiration and narcissistic rivalry moderated changes in relationship satisfaction over time, in a sample of over 5000 romantic dyads, and a subset of dyads in new relationships (one year or less). We found that both one's own and one's partner's narcissistic rivalry were associated with lower relationship satisfaction, consistent with past research showing its association with poor relationship functioning (e.g., Wurst et al. 2017). These effects were stronger for one's own levels of narcissistic rivalry than for one's partner's level. However, contrary to our predictions and prior research showing positive effects of narcissistic admiration, we found no associations between narcissistic admiration and satisfaction or how it changes over time.

### 3.1 Narcissism and Relationship Outcomes Over Time and for Men and Women

Prior research has shown that narcissism may be advantageous in the initial stages of romantic relationships, but detrimental in long‐term relationships (Campbell and Campbell 2009). The NARC framework has been useful in demonstrating how the antagonistic elements of narcissism—narcissistic rivalry—are associated with poor long‐term relationship outcomes, while the agentic elements—narcissistic admiration—are associated with short‐term romantic appeal and even some positive long‐term relationship outcomes (e.g., Wurst et al. 2017). In addition, theory suggests that narcissism may be initially attractive to potential partners, but in the long term lead partners of narcissistic individuals to be particularly dissatisfied (Brunell and Campbell 2011). Consistent with this prior research, we found that narcissistic rivalry was associated with lower satisfaction for both actors and partners, but effects were *larger* for actors. This suggests that narcissistic rivalry shapes how people construct their relationship experiences. Those high in narcissistic rivalry may feel entitled to especially good outcomes, leading to lower satisfaction, regardless of some of the more objective characteristics of their relationship (i.e., them being unresponsive or antagonistic partners). Partners of those high in narcissistic rivalry, on the other hand, may adapt their expectations over time, in response to their partners' narcissistic rivalry.

We did not find negative effects of narcissistic rivalry in the subsample of new relationships, which is consistent with the notion that narcissistic rivalry may take time to cause relationship problems. New relationships are characterized by greater uncertainty (Solomon and Knobloch 2004) and increased efforts at impression management (Leary et al. 1994). The negative, antagonistic behaviors exhibited by those high in narcissistic rivalry may be better concealed or more generously interpreted during the early phase of the relationship.

Contrary to prior research, we found no associations between narcissistic admiration and satisfaction. Perhaps the advantages of narcissistic admiration are more likely to be present during the relationship initiation stage, where agentic self‐presentation matters more than communal responsiveness. It is also possible that the earlier benefits of narcissistic admiration for relationship satisfaction are better captured by more specific relationship outcomes other than satisfaction. Contrary to our hypotheses, neither one's own nor one's partner's narcissism moderated changes in relationship satisfaction—it declined at a similar rate for those higher (or with partners higher) in narcissistic admiration and rivalry.

Further, supplementary analyses using more complete partner data showed that gender had a moderating effect. Female narcissistic rivalry was associated with less satisfaction in longer relationships for men, but the reverse did not occur for the effects of male narcissistic rivalry on female satisfaction. These results were consistent with prior research showing that female, but not male, narcissism is related to lower partner satisfaction (Casale et al. 2020; Gewirtz‐Meydan and Finzi‐Dottan 2018). One reason these gender differences may occur is that women's characteristics and relationship attitudes tend to be more predictive of relationship outcomes than men's (Floyd and Markman 1983; Stanley et al. 2017). Another possibility is that this is more explicitly tied to the interplay between gender and narcissism. Because men are more narcissistic than women (Grijalva et al. 2015), women's narcissism may be seen by partners as more of a norm violation, leading to greater relationship problems (Lamkin et al. 2015).

### 3.2 Do the Effects of Narcissism Vary Across the Relationship Cycle?

Narcissistic rivalry was associated with lower relationship satisfaction, and these effects were invariant over time. Although narcissism did not moderate relationship satisfaction changes over time, other results provided some evidence that the effects of narcissism on satisfaction could depend on relationship length. In our primary analysis, narcissistic rivalry was associated with less satisfaction in the full sample, but not the sample of new relationships. This suggests that the negative effects of narcissistic rivalry may not be present early in a relationship. In addition, exploratory analyses that involved testing the effects of continuously measured relationship length led to similar conclusions, albeit the results were often counterintuitive (e.g., lower narcissistic rivalry was associated with more dramatic declines, although those couples were still more satisfied at the end of the study window).

Our findings have important implications for understanding how narcissism is related to satisfaction at different stages of a romantic relationship. All told, even considering the supplementary analyses, we largely did not find that narcissistic rivalry moderated the decline in relationship satisfaction over 6 years of panel data. One possible explanation for this finding is that the corrosive effects of narcissistic rivalry occur slowly and vary between relationships. One possibility is that the effect of partner narcissistic rivalry on satisfaction is not characterized by a gradual and relatively linear decline, but instead by punctuated declines at specific relationship turning points, such as major conflicts or transitions. Perhaps in some relationships, these effects are seen earlier and in other relationships they are only observed later. In other words, the impact of narcissism on relationships might be more heterogeneous than what we modeled.

These null results might have important implications for how researchers and practitioners characterize the impacts of narcissism on close relationships over time. For example, they raise the possibility that the relational harm caused by having a highly narcissistic partner does not necessarily manifest itself primarily as steeper declines in relationship satisfaction. For example, high levels of narcissistic rivalry could gradually erode partners' self‐esteem or agency, as predicted by self‐determination theory (Sedikides et al. 2019) or lead to increasing negative evaluations of the partner over time, as predicted by some partner perception models. This suggests that the “chocolate cake” characterization of narcissism in relationships (Brunell and Campbell 2011) may not become unsatisfying in a linear way. Rather, it may nonetheless have relational costs that are not well captured by global satisfaction trajectories. Importantly, although these findings may reduce confidence in models proposing that partner narcissistic rivalry uniformly speeds up the decline of satisfaction over time, they do not rule out the possibility of earlier selection effects, nonlinear turning points, or the decline of relational characteristics outcomes other than satisfaction.

Our research design and the assessment timing may have also had implications for our ability to detect whether narcissism was associated with relationship satisfaction changes. For example, those relationships where narcissistic rivalry might be related to the steepest declines would be unlikely to be represented in this sample at all. If narcissistic rivalry led to a steep decline in satisfaction early in the relationship, such as within months or even weeks, these time frames would be too short to capture with this panel data. Thus, narcissistic rivalry might be affecting relationships at a different time scale than what is captured by annual assessments.

Future research should examine the trajectory of relationship satisfaction with measurements that occur frequently during the early stages of a relationship (e.g., every month) to determine if there is an early point in the relationship where narcissistic rivalry becomes associated with relationship difficulties. It is also possible that there are periods of declining satisfaction, which may or may not have coincided with the 6‐year period of this study. This possibility is supported by our exploratory analysis which found that female narcissistic rivalry was associated with lower male satisfaction in longer relationships, a finding that is consistent with the results of Lavner et al. (2016) who found longitudinal effects of wives' entitlement/exploitativeness (the narcissism dimension most closely related to narcissistic rivalry).

## 4 Limitations and Future Directions

The current study had many strengths. It constituted one of the largest studies of narcissism in the context of dyadic close relationships conducted to date. We also leveraged longitudinal data among couples that were both new and more established. Nevertheless, there are some limitations that must be acknowledged.

First, both the narcissism and satisfaction measures were very brief. Although some of our results were consistent with what is seen using longer measures (i.e., the deleterious effects of narcissistic rivalry and the declines in relationship satisfaction), longer and more reliable measures are preferable. Further, relationship satisfaction is just one component of relationship quality, and other components were not included in our study. For example, there is evidence suggesting that people high in narcissism are less invested (Foster 2008) and may also view alternatives to their relationships as more appealing (Campbell and Foster 2002). Further, although lower levels of satisfaction and commitment, and higher quality of alternatives, are associated with reduced commitment and higher dissolution, these risk factors appear to be more dramatic among those higher in narcissism (Foster 2008; Le and Agnew 2003; Le et al. 2010). Broadening the type of outcome measures and formally modeling the mechanisms linking narcissism to relationship outcomes are important to do moving forward. Future research should adopt a more holistic approach to assessing relationship quality, how it changes over time, and how these changes might be different among those higher or lower in NARC components.

Second, while we deliberately restricted some of our analyses to new relationships, our definition of new relationships was based on a cut‐off of 12 months of length. Therefore, some of these dyads were first assessed very close to the beginning of their relationships and others were not. Our supplementary analyses treating relationship length as a continuous variable alleviated this limitation, although the distribution of relationships was not representative of the population. Having predominantly more established relationships likely biased some of our analyses toward these couples, and couples that last longer and are willing to participate in research together are likely different than couples that do not last or are unwilling to participate (Park et al. 2021). This is particularly problematic when trying to make inferences about how these processes operate in short‐ and long‐term relationships. Further, although our study is one of the rare longitudinal tests of actor and partner narcissism on relationship satisfaction over time, we ultimately only had up to 6 years of longitudinal data, and sometimes less. Thus, we were unable to examine the true long‐term trajectory of satisfaction. Future research can address these limitations by studying a more diverse sample of couples followed over longer periods of time, while assessing issues related to selection bias.

## 5 Conclusion

In a large sample of couples followed longitudinally, we found that narcissistic rivalry was associated with lower relationship satisfaction among both individuals and their partners. Despite relationship satisfaction declining on average over time, narcissism did not moderate these changes over time. Supplementary analyses provided conflicting but mostly underwhelming results regarding whether these processes were different among shorter‐ and longer‐term relationships. Future research can more deliberately characterize the influence of narcissism on relationship functioning and longevity.

## Author Contributions

**Gwendolyn Seidman:** conceptualization (lead), writing – original draft (lead), data curation (lead), formal analysis (equal), writing – review and editing (equal), methodology (equal), visualization (equal). **William J. Chopik:** conceptualization (supporting), writing – original draft (supporting), data curation (supporting), formal analysis (equal), writing – review and editing (equal), methodology (equal), visualization (equal).

## Funding

The authors have nothing to report.

## Ethics Statement

pairfam was approved by the ethics committee of the Faculty of Management, Economics and Social Sciences of the University of Cologne.

## Conflicts of Interest

The authors declare no conflicts of interest.

## Supporting information


**Tables S1** jopy70065‐sup‐0001‐TableS1.docx

## Data Availability

Data collected by the German Family Panel pairfam are available to the scientific community at https://www.pairfam.de/en/data/data‐access/.

## Endnotes

^1^
Beginning in Wave 13, narcissism was assessed for both members of the couple. Analyses using these cross‐sectional data, distinguishing on partner sex, are reported in the online supplemental materials, as they fall outside the pre‐registered longitudinal focus of the present manuscript. These results showed that female narcissistic rivalry was associated with lower male satisfaction in longer relationships, but the reverse did not occur for effects of male narcissistic rivalry on female satisfaction.

^2^
These hypothesized were rephrased for clarity. The original language of the pre‐registered hypotheses is available at https://doi.org/10.17605/OSF.IO/KDW5Q.

^3^
This paper uses data from the German Family Panel pairfam, coordinated by Josef Brüderl, Sonja Drobnič, Karsten Hank, Johannes Huinink, Bernhard Nauck, Franz J. Neyer, and Sabine Walper. The study was funded from 2004 to 2022 as a priority program and long‐term project by the German Research Foundation (DFG).

^4^
There were no significant interactions between sex and narcissism dimensions.

^5^
Different operationalizations and modeling of relationship length (e.g., a dichotomous moderating variable such that −1: people in relationships less than a year v. 1: those in relationships more than a year) yielded similar conclusions, albeit one three‐way interaction (between relationship length × rivalry × time) was nonsignificant in some of these models, suggesting it might not be robust to different operationalizations.

^6^
Although this interaction was marginally significant among relatively newer relationships (*b* = −0.030, SE = 0.017, *p* = 0.083, CI: −0.064, 0.004), we decomposed the effects among those high and low in narcissistic rivalry. Decomposing this interaction revealed that relationship satisfaction declines were more dramatic among those higher in narcissistic rivalry (*b* = −0.123, SE = 0.014, *p* < 0.001, CI: −0.151, −0.095) compared to those low in narcissistic rivalry (*b* = −0.085, SE = 0.015, *p* < 0.001, CI: −0.114, −0.056), which is more intuitive and also partially consistent with H2 (although this did not differ between anchors and partners). These results can be seen in Figure S1 (relegated to the supplement because the interaction was marginally significant). Thus, relationship length did matter, albeit in somewhat obscure ways. Among new relationships, narcissistic rivalry was particularly damaging for relationship satisfaction over time. However, among more established couples, the role of narcissistic rivalry is a bit ambiguous.
