# Supplementary material for: From Spark to Strain? Changes in Relationship Satisfaction as a Function of Narcissistic Admiration and Rivalry
Source: J Pers. 2026 Mar 26;94(6):1045–56. doi: 10.1111/jopy.70065 (PMC13768732; doi:10.1111/jopy.70065)
Supplement: Supplementary file 1 [file JOPY-94-1045-s001.docx]

**Integrating Partner Reports of Narcissism in Wave 13**

In the main analyses, we used anchor status as the distinguishing variable because narcissism was only assessed for anchors at wave 9. However, beginning in Wave 13, *both* anchors and partners completed the narcissism measure, effectively doubling the amount of narcissism information available to us. We included these data here for transparency purposes in that there was additional partner narcissism data available at a later wave. While this did not allow us to examine longitudinal effects of both partners’ narcissism using both members of each couple, it did give us the opportunity to more robustly test for sex differences in actor/partner effects of narcissism and relationship length cross-sectionally in Wave 13 (a point of contention in previous research). Therefore, we ran an APIM using only the Wave 13 data, distinguishing on sex (coded as -1 for male and 1 for female). We tested the effects of relationship length, actor and partner narcissistic admiration, actor and partner narcissistic rivalry and interactions between relationship length and actor and partner narcissism on relationship satisfaction.

Supplemental Table 2 displays estimates, standard errors, confidence intervals and significance testing results for a single-intercept model. Overall, regardless of sex, actor narcissistic rivalry was associated with lower satisfaction, and actor narcissistic admiration was associated with higher satisfaction (partially confirming H1 and H2, albeit cross-sectionally). While partner narcissistic admiration was not significantly associated with satisfaction, partner narcissistic rivalry was associated with lower satisfaction. There were no significant interactions between relationship length and actor or partner effects for either narcissism dimension. This suggests that the actor and partner effects of both narcissism variables on relationship satisfaction did not differ as a function of relationship length.

However, several significant three-way interactions supported the notion that there were sex differences in the effects of narcissism as a function of relationship length. There was a marginally significant actor narcissistic admiration X relationship length X sex interaction and there were significant interactions between relationship length, sex, and partner effects for both narcissism dimensions.

To further explore these three-way interactions, we ran a dual-intercept version of the APIM, estimating separate effects for males and females (See Supplemental Table 3 for results).^[1]^ Female narcissistic admiration was marginally positively associated with marginally male satisfaction in longer relationships, but effects of male narcissistic admiration on female satisfaction did not differ as a function of length. Female narcissistic rivalry was significantly negatively associated with male satisfaction in longer relationships, but effects of male narcissistic rivalry on female satisfaction did not differ as function of length. All told, there is some evidence that female narcissism is a stronger predictor of both their and their male partner’s relationship satisfaction than men’s narcissism. Given the large number of tests and the relatively small interaction effects (and *p*-values close to *p* = .05), these exploratory tests should be interpreted with caution, as they would likely become non-significant with even a modest alpha-level correction.

**Supplemental Table 2**

*Estimates for Single-Intercept Actor Partner Interdependence Model Distinguished by Sex for Wave 13*

| *Fixed Effects* | *b* | *SE* | *t* | *p* | *CI* |
| --- | --- | --- | --- | --- | --- |
| Intercept | 8.296 | .031 | 267.35 | <.001 | (8.236, 8.357) |
| Sex | -.066 | .019 | -3.452 | .001 | (-.103, -.028) |
| Actor narcissistic rivalry | -.320 | .044 | -7.315 | <.001 | (-.406, -.234) |
| Actor narcissistic admiration | .065 | .032 | 2.058 | .040 | (.003, .127) |
| Partner narcissistic rivalry | -.154 | .043 | -3.578 | <.001 | (-.238, -.070) |
| Partner narcissistic admiration | .038 | .031 | 1.230 | .219 | (-.023, .100) |
| Relationship length | -.0002 | .001 | -.228 | .820 | (-.002, .002) |
| Actor narcissistic rivalry x length | -.0007 | .0004 | -1.495 | .135 | (-.002, .0002) |
| Actor narcissistic admiration x length | -.00002 | .0003 | -.057 | .954 | (-.0006, .0006) |
| Partner narcissistic rivalry x length | -.0003 | .0004 | -.739 | .460 | (-.001, .0005) |
| Partner narcissistic admiration x length | .00003 | .0003 | .090 | .929 | (-.006, .0006) |
| Actor narcissistic rivalry x sex | -.050 | .047 | -1.084 | .278 | (-.142, .041) |
| Actor narcissistic admiration x sex | .040 | .033 | 1.191 | .234 | (-.026, .105) |
| Partner narcissistic rivalry x sex | .063 | .046 | 1.383 | .167 | (-.026, .153) |
| Partner narcissistic admiration x sex | -.008 | .033 | -.241 | .810 | (-.073, .057) |
| Length x sex | .0001 | .0004 | .622 | .534 | (-.0002, .0002) |
| Actor narcissistic rivalry x length x sex | -.0007 | .0005 | -1.621 | .105 | (-.002, .0001) |
| Actor narcissistic admiration x length x sex | .0006 | .0003 | 1.747 | .081 | (-.00007, .001) |
| Partner narcissistic rivalry x length x sex | .001 | .0004 | 2.485 | .013 | (.0002, .002) |
| Partner narcissistic admiration x length x sex | -.0007 | .0003 | -2.284 | .022 | (-.001, -.0001) |
| *Goodness-of-fit indices* |  |  |  |  |  |
| AIC | 15497 |  |  |  |  |
| -2LL | 15491 |  |  |  |  |

*Note.* Sex is coded as -1 for male and 1 for female.

**Supplemental Table 3**

*Estimates for Dual-Intercept Actor Partner Interdependence Model Distinguished by Sex for Wave 13*

| *Fixed Effects* | *b* | *SE* | *t* | *p* | *CI* |
| --- | --- | --- | --- | --- | --- |
| Male intercept | 8.362 | .035 | 242.278 | <.001 | (8.295, 8.430) |
| Female Intercept | 8.231 | .038 | 215.22 | <.001 | (8.156, 8.306) |
| Relationship length on male satisfaction | -.002 | .0003 | -5.532 | <.001 | (-.003, -.001) |
| Relationship length on female satisfaction | -.002 | .0003 | -4.372 | <.001 | (-.002, .0009) |
| Male actor narcissistic admiration on male satisfaction | .025 | .041 | .613 | .540 | (-.055, .106) |
| Female narcissistic admiration on female satisfaction | .105 | .050 | 2.078 | .038 | (.006, .204) |
| Male narcissistic rivalry on male satisfaction | -.270 | .055 | -4.904 | <.001 | (-.378, -.262) |
| Female narcissistic rivalry on female satisfaction | -.371 | .072 | -5.172 | <.001 | (-.511, -.230) |
| Female narcissistic admiration on male satisfaction | .046 | .046 | 1.019 | .309 | (-.043, .136) |
| Male partner narcissistic admiration on female satisfaction | .030 | .046 | .669 | .504 | (-.959, .0120) |
| Female partner narcissistic rivalry on male satisfaction | -.217 | .065 | -3.359 | .001 | (-.344, -.090) |
| Male partner narcissistic rivalry on female satisfaction | -.091 | .061 | -1.485 | .138 | (-.0210, .029) |
| Male narcissistic admiration X length on male satisfaction | -.0006 | .0004 | -1.472 | .141 | (-.001, .0002) |
| Female narcissistic admiration X length on female satisfaction | .0005 | .0005 | 1.116 | .264 | (-.0004, .002) |
| Male narcissistic rivalry X length on male satisfaction | -.0001 | .0005 | .171 | .864 | (-.0009, .001) |
| Female narcissistic rivalry x length on female satisfaction | -.001 | .0007 | -1.915 | .056 | (-.003, .00003) |
| Female narcissistic admiration X length on male satisfaction | .0008 | .0004 | 1.713 | .087 | (-.0001, .002) |
| Male narcissistic admiration X length on female satisfaction | -.0007 | .0004 | -1.605 | .109 | (-.002, .0002) |
| Female narcissistic rivalry X length on male satisfaction | -.001 | .0007 | -2.171 | .030 | (-.003, -.0001) |
| Male narcissistic rivalry X length on female satisfaction | .001 | .0006 | 1.3840 | .167 | (-.0003, .002) |

| **Supplementary Table 1** |  |  |  |  |  |
| --- | --- | --- | --- | --- | --- |
| *Examining Relationship Length as a Continuous Moderator* |  |  |  |  |  |
| Fixed Effects | *b* | *SE* | *t* | *p* | *95% CI* |
| Intercept | 8.020 | .028 | 283.548 | <.001 | (7.965, 8.075) |
| Anchor status | .126 | .012 | 10.235 | <.001 | (.102, .150) |
| Time | -.058 | .006 | -9.103 | <.001 | (-.071, -.046) |
| Narcissistic rivalry | -.298 | .035 | -8.495 | <.001 | (-.367, -.229) |
| Narcissistic admiration | .046 | .026 | 1.758 | .079 | (-.005, .098) |
| Relationship Length | .001 | .0003 | 2.541 | .011 | (.0001, .001) |
| Time * Anchor status | .005 | .005 | 1.076 | .282 | (-.004, .015) |
| Narcissistic rivalry * Anchor status | .067 | .023 | 2.986 | .003 | (.023, .112) |
| Narcissistic admiration * Anchor status | .0002 | .017 | .012 | .991 | (-.033, .034) |
| Anchor status * Relationship Length | -.00007 | .0001 | -.520 | .603 | (-.0003, .0002) |
| Time * Narcissistic rivalry | .005 | .012 | .454 | .650 | (-.018, .029) |
| Time * Narcissistic admiration | -.009 | .009 | -1.033 | .302 | (-.027, .008) |
| **Time * Relationship Length** | **.0005** | **.00007** | **7.142** | **<.001** | **(.0003, .001)** |
| **Narcissistic rivalry * Relationship Length** | **-.001** | **.0004** | **-2.290** | **.022** | **(-.002, -.0001)** |
| Narcissistic admiration * Relationship Length | .0001 | .0003 | .364 | .716 | (-.0005, .001) |
| Time * Narcissistic rivalry * Anchor status | -.020 | .009 | -2.129 | .033 | (-.038, -.002) |
| Time * Narcissistic admiration * Anchor status | -.003 | .007 | -.381 | .704 | (-.016, .011) |
| Time * Anchor status * Relationship Length | -.00004 | .0001 | -.825 | .410 | (-.0001, .00006) |
| Narcissistic rivalry * Anchor status * Relationship Length | -.0001 | .0002 | -.281 | .779 | (-.001, .0004) |
| Narcissistic admiration * Anchor status * Relationship Length | -.0001 | .0002 | -.366 | .715 | (-.0004, .0003) |
| **Time * Narcissistic rivalry * Relationship Length** | **.0004** | **.0001** | **2.876** | **.004** | **(.0001, .001)** |
| Time * Narcissistic admiration * Relationship Length | -.0001 | .0001 | -.816 | .414 | (-.0003, .0001) |
| Time * Narcissistic rivalry * Anchor status * Relationship Length | .0001 | .0001 | .693 | .489 | (-.0001, .0003) |
| Time * Narcissistic admiration * Anchor status * Relationship Length | .0001 | .0001 | 1.089 | .276 | (-.00007, .0002) |
| Age | -.040 | .003 | -14.362 | <.001 | (-.045, -.034) |
| Sex | -.082 | .037 | -2.197 | .028 | (-.154, -.009) |
| *Note.* Significant moderation effects for relationship length are bolded. |  |  |  |  |  |

**Supplementary Figure 1**

*Narcissistic Rivalry and Relationship Satisfaction Changes among those in Newer Relationships*

1. Although there were no sex differences in overall partner effects from the single-intercept model, for females, their own narcissistic admiration was positively associated with satisfaction, but there was no association for males (i.e., the effects of narcissistic admiration on relationship satisfaction did not differ between men and women [in effect size], even though the effect for women was significantly different from zero). Further, female narcissistic rivalry was associated with lower satisfaction for males, but male narcissistic rivalry did not predict female satisfaction.
